# A High-Throughput Circular Tumor Cell Sorting Chip with Trapezoidal Cross Section

**DOI:** 10.3390/s24113552

**Published:** 2024-05-31

**Authors:** Shijie Lu, Ding Ma, Xianqiang Mi

**Affiliations:** 1 School of Microelectronics, Shanghai University, 20 Chengzhong Road, Shanghai 201899, China; 994613623@shu.edu.cn; 2 National Key Laboratory of Materials for Integrated Circuits, Shanghai Institute of Microsystem and Information Technology, Chinese Academy of Sciences, 865 Changning Road, Shanghai 200050, China; m18211655237@163.com; 3 University of Chinese Academy of Sciences, Beijing 100049, China

**Keywords:** high throughput, microfluidic, CTC, sorting

## Abstract

Circulating tumor cells are typically found in the peripheral blood of patients, offering a crucial pathway for the early diagnosis and prediction of cancer. Traditional methods for early cancer diagnosis are inefficient and inaccurate, making it difficult to isolate tumor cells from a large number of cells. In this paper, a new spiral microfluidic chip with asymmetric cross-section is proposed for rapid, high-throughput, label-free enrichment of CTCs in peripheral blood. A mold of the desired flow channel structure was prepared and inverted to make a trapezoidal cross-section using a micro-nanotechnology process of 3D printing. After a systematic study of how flow rate, channel width, and particle concentration affect the performance of the device, we utilized the device to simulate cell sorting of 6 μm, 15 μm, and 25 μm PS (Polystyrene) particles, and the separation efficiency and separation purity of 25 μm PS particles reached 98.3% and 96.4%. On this basis, we realize the enrichment of a large number of CTCs in diluted whole blood (5 mL). The results show that the separation efficiency of A549 was 88.9% and the separation purity was 96.4% at a high throughput of 1400 μL/min. In conclusion, we believe that the developed method is relevant for efficient recovery from whole blood and beneficial for future automated clinical analysis.

## 1. Introduction

Cancer remains one of the leading causes of human mortality worldwide. Circulating tumor cells (CTCs) are rare cells released from primary tumors that spread to other organs through the peripheral blood circulation and infect healthy tissues which are essential for metastatic studies and early diagnosis of cancer [1,2]. The isolation, enumeration, and analysis of CTCs are important for clinical applications such as early cancer diagnosis, progression, and assessing the effectiveness of drugs in personalized cancer therapy [3,4]. Collecting and detecting CTCs in the blood is challenging due to their rarity. 

Microfluidic technologies for CTC separations are of interest because of their low cost, rapid operation, and high throughput. Methods for efficient isolation of CTCs [5,6,7,8] can be categorized into physical sorting and biochemical sorting. Physical methods [9,10,11], including active and passive sorting, mainly utilize the physical properties of tumor cells, such as size density, electric charge, etc. [12,13]. Active physical sorting can achieve high separation efficiency and accuracy, but the need for complex external field assistance (e.g., optical [14], electrical [15,16,17], magnetic [18], or acoustic [19]) greatly raises the cost and inconvenience. To address this challenge, methods for passive separation of CTCs have been introduced [20]. Passive sorting methods rely only on the structure to induce the particles to display different flow states when subjected to hydrodynamic fluids to achieve sorting. According to the different effects produced by the particles, passive sorting techniques can be classified into size-based sorting [21], inertial force sorting [22], deterministic lateral displacement sorting [23], and elastic sorting [24]. 

Inertial sorting is gradually becoming the mainstream of passive sorting due to its high throughput and convenient operation. The working principle of inertial cell sorting [25,26] is that, under certain fluid conditions, the suspended particles in the fluid experience wall-induced lift, shear-induced lift, and other inertial forces perpendicular to the direction of particles through the channel, resulting in lateral migration. Since particles of different sizes are subjected to different lateral migration forces, they eventually equilibrate to different positions in the micro-channel to achieve focusing, sorting, and enrichment of mixed particles [27,28,29]. Using this principle, Bhagat et al. [30] designed an elongated straight channel and a trident exit structure, which succeeded in separating large and small particles, but the results were poor. 

Geometry (topology) [31] would influence the performance of inertial CTCS sorting. Based on the geometry, the current inertial sorting channels include spiral channels [32,33,34,35], wave channels [36], and contraction/expansion channels [37]. One of the most widely used geometries for inertial sorting is the spiral channel. In spiral microfluidic chips, the fluid experiences centrifugal acceleration in the radial outward direction, leading to the formation of two counter-rotating vortices known as Dean vortices [38]. In the presence of a Dean vortex, particles within the flow channel generate a Dean drag force. Cells are subjected to a combination of inertial and Dean traction forces in the spiral micro-channels. This causes larger target cells to move to the inner wall, while smaller cells are dispersed and move along the flow line, ultimately enabling the sorting of cells of different sizes. In 2008, a spiral microfluidic chip based on Dean flow fractionation (DFF). which could separate 1.9 μm PS particles was initially presented by Bhagat et al. [39]. Then, with conditions of mobility continuously being optimized, they successfully isolated CTCs in 2013 [40]. Sun et al. [41,42] designed a double spiral structure that could separate blood-doped MCF-7 or HeLa tumor cells at a flux of 20 mL/h with a separation efficiency of 96.77%.

The cross-sectional shape of the micro-channels [43] is important factor influencing the performance of spiral microfluidic chips. Because of the optimized cross-section, there is a greater disparity of forces on the different sizes of cells within the spiral channel, and the separation becomes even more excellent [44]. Warkiani, M., Khoo, B., and Wu, L. et al. [45] designed a two-ring structure with dual inlets and rectangular cross-section, resulting in ≥85% cell recovery for a wide range of cancer cell lines. However, its throughput still needs improvement. Abdulla et al. [46] isolated two types of CTCs (A549 cells and MCF-7 cells) using a microfluidic device consisting of two-ring spiral channels and a zigzag channel. Similarly, Rzhevskiy et al. [47] used a five-ring spiral micro-channel with a trapezoidal cross-sectional channel to isolate prostate cancer cells (PCa) from urine with a separation efficiency of 86%. 

**Figure 1:**
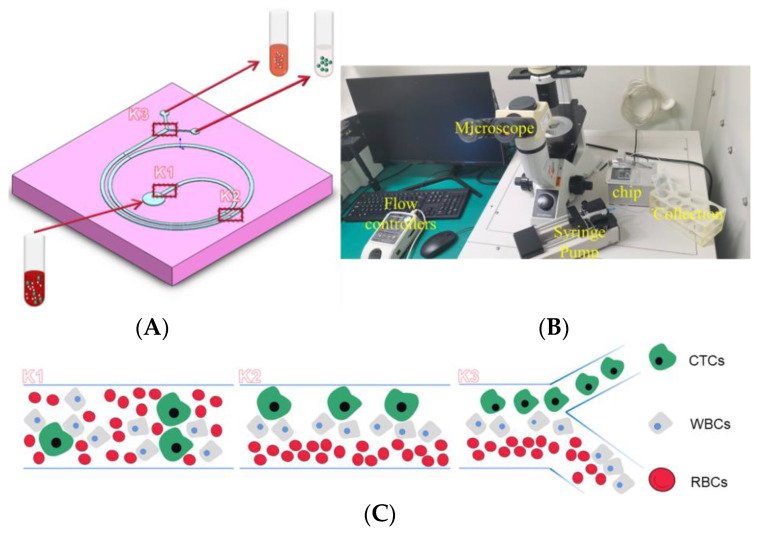
Sorting principle and basic design of the chip. (**A**). Basic design and workflow; (**B**). finished chip; (**C**). schematic of sorting principle (circulating tumor cells—CTCs, white blood cells—WBCs, red blood cells—RBCs).

In this paper, we propose a single-inlet microfluidic chip device with a spiral structure and a trapezoidal cross-section as shown in Figure 1A. The system loaded with a trapezoidal cross-sectioned two-ring spiral chip is shown in Figure 1B. This device is used for ultra-high-throughput sorting of CTCs and can separate 5 mL of whole blood in less than 4 min. The specific separation process is shown in Figure 1C; the liquid mixed with different cell sizes flows at the position of K1, the distribution of different cell sizes in the K2 fluid changes, and finally complete separation is achieved at the outlet of the channel K3. By studying the flow rate and micro-channel width to length ratio, a cell sorting efficiency of 88.9% was achieved for A549 cells. In addition, the simple separation principle using only fluidic forces enables cell recovery with minimal damage to the cells. A novel preparation method is used in this paper which greatly reduces the cycle time and cost of microfluidic chip optimization. Our device is expected to have future applications for automated detection and analysis of CTC in whole blood.

## 2. Materials and Methods

### 2.1. Principle of Operation

Dean drag in the opposite direction to the inertial lift. The force exerted by the fluid in the micro-channel determines the final equilibrium position of the particles. The trapezoidal cross-section primarily affected particles in the micro-channels by inertial lift forces, including shear gradient lift ($F_{s}$) and wall-induced lift ($F_{w}$). 

The particles experience shear gradient lift ($F_{s}$) due to the parabolic rates distribution in the Poisson lobe flow, causing them to be pushed towards the channel wall [48], then experience a second dominant force known as wall-induced lift $F_{w}$, which drags them away from the wall and causes them to flow near the centerline of the channel [49]. Thus, the equilibrium position of the particle in the channel cross-section is determined by the mutual balance of these two opposing forces. The net inertial lift $F_{L}$, which is the combined force of $F_{s}$ and $F_{w}$ acting on the particle, is calculated as follows [50,51]:

(1) $$F_{L}=C_{L}\frac{\rho U_{max}a_{p}^{4}}{D_{h}^{2}}$$

(2) $$F_{S}\propto \frac{\rho U^{2}a^{3}}{H}$$

(3) $$F_{W}\propto \frac{\rho U^{2}a^{6}}{H^{4}}$$

where $C_{L}$ is a lift coefficient related to particle position and cross-sectional fluid rate, ρ is the fluid density, $U_{max}$ is the maximum fluid rates, $a_{p}$ is the particle diameter, and $D_{h}$ is the channel hydraulic diameter [52,53]. 

The channel’s curved spiral structure primarily affects particles in the micro-channels via two counter-rotating flows known as Dean vortices [50] which exerts a Dean flow force on the particle, expressed as follows [54]:

(4) $$F_{D}=5.4\times 10^{-4}\pi \mu {De}^{1.63}a_{p}$$

Here, μ represents the dynamic viscosity of the liquid, and De, the dimensionless Dean number, is defined as follows [55,56]:

(5) $$De=\frac{\rho \bar{U}D_{h}}{\mu }\times \sqrt{\frac{D_{h}}{2R}}=Re\times \sqrt{\frac{D_{h}}{2R}}$$

R is the channel radius of curvature, U is the mean fluid rates, and Re is the channel Reynolds number.

In a Dean vortex, the particle is subjected to Dean drag in the opposite direction to the inertial lift. Since the Dean flow force ($F_{D}$) is not as sensitive to particle diameter as the inertial lift force ($F_{L}$∝$a_{p}^{4}$, $F_{D}$∝$a_{p}$), smaller particles tend to concentrate more towards the center of the Dean vortex close to the outer wall under the combined action of the two forces [48,57]. In addition, the larger particles are focused near the inner wall by the inertial lift force.

**Figure 2:**
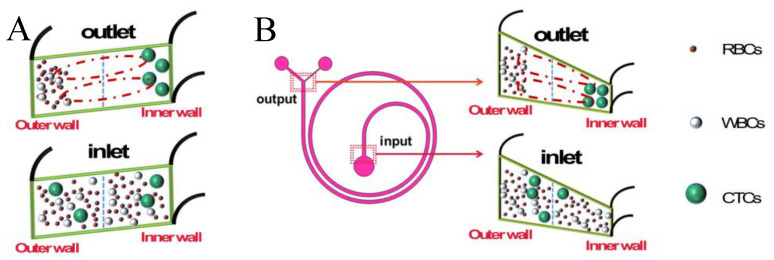
Distribution of particles in the channel before and after fluid action. (**A**). Distribution of particles before and after force in the rectangular channel; (**B**). Distribution of particles before and after force in the trapezoidal channel.

Figure 2 shows the distribution of particles in the rectangular and trapezoidal cross-section channels before and after being subjected to the fluid force. The trapezoidal cross-section microfluidic chip designed in this paper is shown in Figure 2B. The Dean vortex generated by the trapezoidal cross-section channel has a more pronounced focusing effect on the particles than the typical rectangular cross-section, as shown in Figure 2A. In the Dean vortex, smaller-sized particles (RBCs, WBCs) are focused towards the vicinity of the outer wall of the micro-channel and eventually ejected from the outer outlet, while larger particles (CTCs) equilibrate near the inner wall and are collected by the inner outlet. Figure 2B shows how particles in a channel with a trapezoidal cross-section are affected by inertial lift and drag. According to Figure 2B with Equations (1) and (2), it can be seen that the larger sized cells (CTCs) that pass into the chip are concentrated in the inner wall, while the smaller cells (WBCs and RBCs) and debris are located near the outer wall.

### 2.2. Device Design and Fabrication

The polymer microfluidic chip consists of two-loop spiral channels for cell separation based on different sizes. The radius of each spiral channel loop was gradually increased from 4 mm to 10 mm. The optimal structure of the chip channel was designed based on a previous study on the effect of different trapezoidal cross-sections of spiral channels on the inertial separation performance [58]. The cross section of the spiral channel was set as a right-angle trapezoid with an inner height of 75 μm and an outer height of 150 μm. The channel width was determined to be 500 μm.

Unlike the traditional soft lithography process, this paper proposes a new process to prepare trapezoidal cross-section chips using a combination of 3D printing and soft lithography. In order to fabricate micro-channels with trapezoidal cross-section, ABS molds are prepared by 3D printing to meet specific geometrical requirements. We use ABS as the printing ink, supplemented by resin, which gives our molds a higher degree of precision. This allows us to fabricate trapezoidal cross-section structures with a target inclination. The tolerance of the template molds against the CAD model was ±10 μm according to the capability of the 3D printer (Photonic Professional GT2, Nanoscribe Company, Karlsruhe, Germany). Subsequently, the PDMS prepolymer base and the curing agent were mixed in a ratio of 10:1. The PDMS mixture was then degassed using a desiccator for 120 min, poured onto prepared molds, and cured in an oven at 70 °C for 8 h. When the molds were stripped, the inlets and outlets were punched with 1 mm and 3 mm diameter punches. The microscope slides and PDMS channels were then cleaned with isopropyl alcohol, deionized water, and clear tape. After nitrogen drying, the PDMS channels and slides were bonded in an oxygen plasma device for 60 s with intermittent oxygen injection. To form a closed micro-channel structure, the treated surfaces were pressed together to form stable bonds. Finally, the PDMS microfluidic chip prepared for this study was cut at different positions of the main channel and the cross-section was observed under a microscope (IX71, OLYMPUS Corporation, Tokyo, Japan) to evaluate the dimensions of the device.

### 2.3. Numerical Simulation Settings

Based on previous work, in our in-house work in the microchip lab, the carrier fluid has been considered as water as in diluted whole blood, and the density and dynamic viscosity of diluted whole blood were considered as ρ = 1000 kg/m^3^ and μ = 0.001 Pa·s [59]. The whole numerical simulation consists of two modules, the laminar flow module for establishing the flow field environment and the particle tracking module for tracking the movement of CTCs and other cells within the micro-channels.

We use a laminar flow based module to create the flow field in the micro-channel. In this module, the Navier–Stokes equations and the continuity equations are used as controlling equations. Considering single-phase Newtonian (incompressible) laminar flow, these equations are as follows [60]:

(6) $$\rho \left(u\cdot \nabla \right)=\nabla \left[-pI+K\right]$$

(7) ρ∇·u=0

(8) $$K=\left(\nabla u+{\left(\nabla u\right)}^{T}\right)\mu$$

Here, ρ and u are denoted as pressure and rates vectors, respectively. F is the volumetric force vector, and u and ρ are the kinetic viscosity and kinetic density, respectively. Temperature is denoted as temperature by K. Initially, the inlet flow rates of the blood sample were taken to be 0 m/s. The boundary condition for the pressure at the outlet is set to 0 Pa. The Navier–Stokes equations are solved using finite element simulation and the default fluid discretization parameters are used for the setup. In this paper, a no-slip boundary condition is set for the micro-channel wall.

The particle tracking module was used to track the location of particles (blood cells and CTCs) within the micro-channel. A total of 340 cells were injected at the lab-on-a-chip inlet shown in Figure 1, including 40 A549s, 100 WBCs, and 200 RBCs. Here, the drag force (Law of Drag: Stokes) and the wall-induced lift force were applied to the cells. Their governing equations have been given in Equations (1) and (2). The module also employs several boundary conditions, such as setting the micro-channel wall condition as elastic and the exit wall condition as frozen. The module is solved by Euler’s method using a time-dependent solver. Here, the solution of the laminar flow module is used as the rate input for particle or cell position tracking. Numerical simulations were performed using the following system specifications: Lenovo R720-15I7BN with Intel(R) Core(TM) i5-7300HQ CPU @ 2.50 GHz processor and 8.00 GB RAM (Lenovo Company, Beijing, China).

### 2.4. Sample Preparation

Solutions of polystyrene particles with diameters of 6, 15, and 25 μm were used to simulate the separation and aggregation of blood cells and tumor cells. The validation experiments of fluorescent particles were performed using 6 μm and 25 μm fluorescent polystyrene microspheres (Zhichuan Technology Company, Suzhou, China) with a mass fraction of 25 mg/mL. Solutions of ×2, ×5, ×10, and ×50 times dilutions of the particles were prepared. The diluent was mainly phosphate buffer containing 0.1% Tween 20 (Sangon Biotech, Shanghai, China), which could prevent the particles from adhering to each other. The polystyrene particle solution was sonicated for 15–30 min to reduce particle agglomeration and, consequently, minimize the effect of inter-particle interactions on particle sorting.

The A549 cell line was cultured in flasks with DMEM/F12 medium (Sangon Biotech, Shanghai, China). The medium was supplemented with 10% (penicillin/streptomycin) FBS to obtain complete medium for A549 growth. After incubation, the cells were placed in a humidified environment at 37 °C and 5% CO_2_. Afterwards, the medium was replaced with fresh medium every two days, and this process was continued until the cells reached the third generation.

After obtaining a sufficient number of A549 cells, these cells were counted using a cell counter, then the A549 cells were mixed with diluted whole blood from healthy volunteers at a specific ratio of 1:1000. Finally, the cell samples in the above proportions were injected into the device through the air inlet.

In addition, DAPI staining was used to aid in the visibility of target cells (A549) after isolation, resulting in more accurate cell counts for exit. To accomplish this, cells were first fixed in paraformaldehyde (PFA). Then, cells were permeabilized with Triton X-100 (Sangon Biotech, Shanghai, China) in PBS and then stained with DAPI.

### 2.5. Experimental Setup

The experiment was performed on a complete microfluidic setup that included a syringe pump, fluorescence microscope, collection tube, 5 mL syringe, and camera. Prepared samples were injected into the microfluidic device via a syringe pump, and the outlet flow was then collected in a microtube. A microscope was used to capture and monitor the fluid flow of the sample through the micro-channel. It is important to note that each of these devices was used multiple times in the tests mentioned above.

## 3. Results and Discussion

### 3.1. Simulation Results and Discussion

The effects of different flow rates and widths of trapezoidal cross-section spiral micro-channels on the separation performance of the chip. It also suggested appropriate design parameters and their influence on the separation of CTCs. The widely used two-turn rectangular spiral micro-channel chips designed by previous authors were compared under the same parameter conditions, and the variation of the separation efficiency of the chip for CTCs in cell-mixed samples was numerically calculated and plotted. All numerical simulations were derived from finite element simulation software.

**Figure 3:**
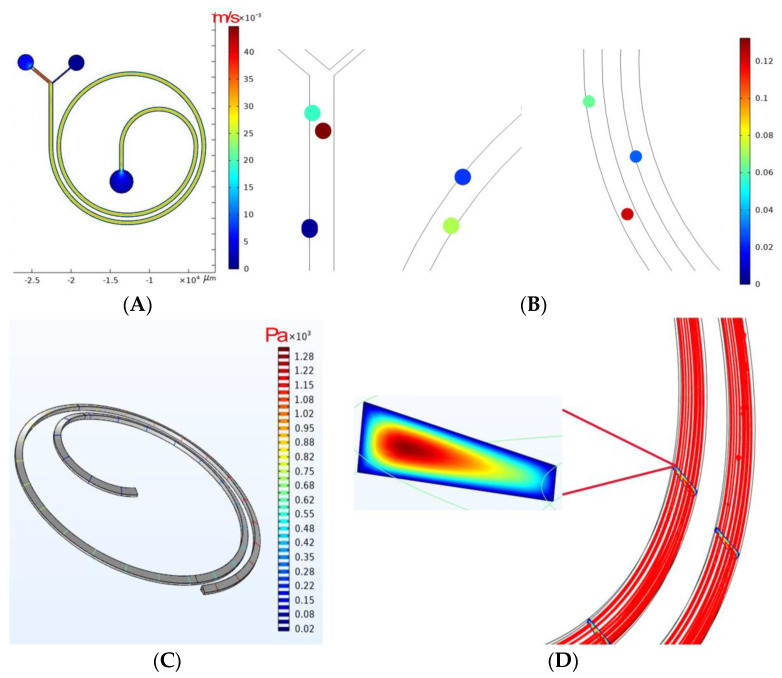
In-channel fluid simulation with particle tracking. (**A**). Flow rate distribution; (**B**). particle tracking distribution; (**C**). 3D in-channel pressure distribution; (**D**). 3DStreamline distribution.

Initially, the flow rate at the inlet of the chip was set to 500 μL/min. The planar distribution of the fluid flow rate in the spiral micro-channel with trapezoidal cross-section is shown in Figure 3A. It can be seen that the fluid flow rate in the micro-channel is uniformly distributed and changes at the outlet. This indicates that the fluid in the channel strongly affects the outer wall of the channel near the outlet. Meanwhile, the pressure inside the channel is shown in Figure 3C, showing a continuous decrease along the flow channel while remaining uniformly distributed. This suggests that the fluid flow and Dean vortex within the channel are stable. With the injection of particles, the flow lines inside the micro-channel, as shown in Figure 3D, are denser near the outer wall of the channel, mirroring the results in Figure 3A, indicating that the fluid inside the micro-channel migrates cells with small sizes, such as RBCs, to the outer wall of the channel. After the simulation was carried out for a period of time, the particle distribution within the micro-channel is shown in Figure 3B. It can be seen that the larger A549 are subjected to inertial lift forces stronger than Dean flow forces. As a result, they do not undergo Dean migration and stay at the inner wall of the channel. The smaller RBCs and WBCs, on the other hand, are subject to the inertial lift force weaker than the Dean flow force and as such undergo Dean migration themselves and are carried to the outer wall of the micro-channel. The result is consistent with the theory.

The separation of A549 from normal blood cells, WBCs, and RBCs at different flow rates was analyzed using a two-turn trapezoidal spiral micro-channel chip. Performance was analyzed using the purity of cell separation given in Equation (7) and separation efficiency given in Equation (8).

(9) $$Separation\;purity={\left(\frac{target\;samples}{target\;samples+nontarget\;samples}\right)}_{in\;collection}$$

(10) $$Separation\;efficiency=\frac{{\left(target\;samples\right)}_{in\;collection}}{{\left(target\;samples\right)}_{inlet}}$$

**Figure 4:**
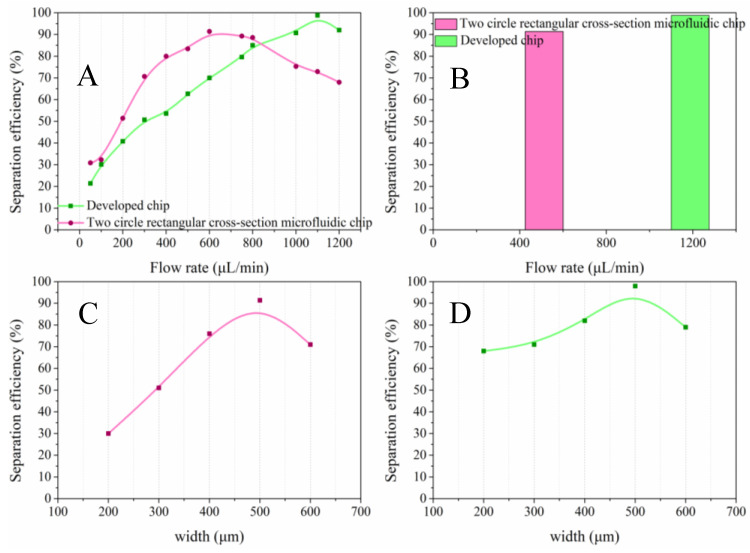
Results of simulation calculation of separation efficiency. (**A**). Comparison of flow rate influence on separation efficiency between mainstream two-turn rectangular and developed trapezoidal spiral micro-channel chip; (**B**). optimal efficiencies of the two types of chips; (**C**). influence of the channel width on the performance of the mainstream chip; (**D**). influence of the channel width on the performance of the trapezoidal spiral chip.

The separation performance of A549 cell separation efficiency at different flow rates has been plotted and is shown in Figure 4. Figure 4A compares the capture capacity of A549 cells from the current mainstream two-turn rectangular spiral micro-channel chip and our designed two-turn trapezoidal spiral micro-channel chip. It demonstrates the impact of flow rate on the separation performance. The results showed that the capture efficiency of the two chips increased with increasing flow rate. The mainstream two-turn rectangular spiral micro-channel chip peaked at 600 μL/min, while the two-turn trapezoidal spiral micro-channel chip peaked at 1100 μL/min. As shown in Figure 4B, the accuracy is 91.4% for the former and 98.8% for the latter. It can be seen that the chip surpasses the mainstream two-turn helical microfluidic chip in terms of separation efficiency and has a higher throughput. Based on this result, the impact of the main channel width on sorting performance is investigated alongside the flow rate that achieves the highest recovery efficiency. As can be seen in Figure 4C,D the cell recovery efficiency increases continuously with increasing width and peaks at a width of 500 μm for both chips. Based on the previous results, to prepare the two-turn trapezoidal spiral micro-channel chip with the best performance, the structural parameter of the main channel width of 500 μm was selected and a flow rate of 1100 μL/min was applied.

### 3.2. Particle Sorting Experiment

Based on the simulation results, a 500 μm width trapezoidal spiral micro-channel chip was prepared as a way to explore the optimal flow rate and concentration, and the blended PS particles of various particle sizes were passed into the prepared size-optimized chips.

**Figure 5:**
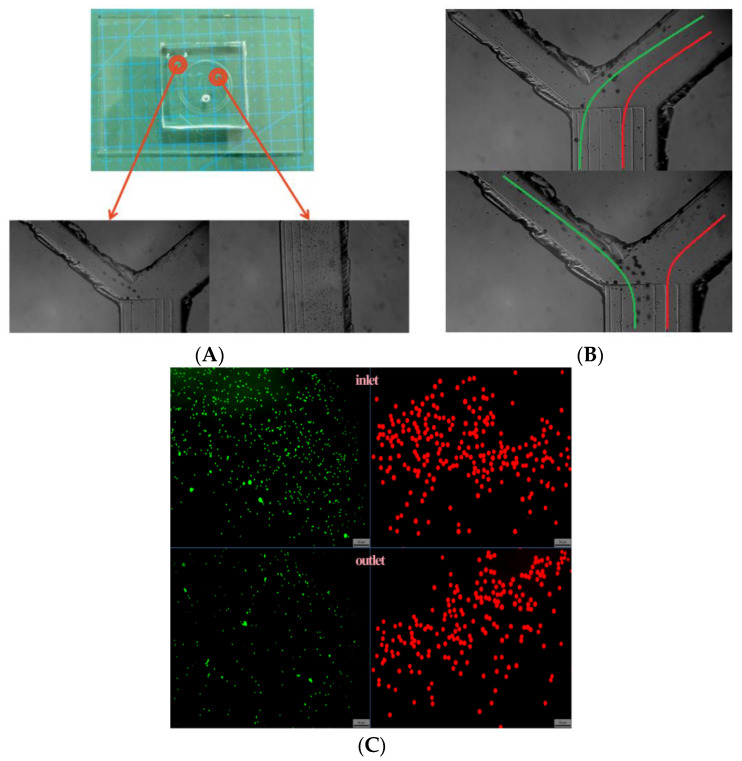
Chip inlet and outlet particle tracking and sorting effect. (**A**). Distribution of particles in the channel and at the outlet (scale bar: 500 μm); (**B**). particle distribution to form the corresponding streamlines (6–10 μm particle streamlines in red, 25 μm particle streamlines in green) (scale bar: 150 μm); (**C**). distribution of particles in the sample at the inlet and outlet (scale bar: 50 μm).

After passing through the inlet, the mixed solution containing 6–15 μm and 25 μm particles gradually separates from its initial state in the middle part of the channel under the action of Dean and lifting forces and then concentrates again near the outlet. At this point, particles of 25 μm in size collect on the inner wall of the channel, while the other particles gather on the outer side of the channel (as shown in Figure 5).

As the flow rate increased from 100 μL/min to 1000 μL/min, the balance between the Dean force and lifting force changed, and the streamlines formed by the focusing of 25 µm particles changed from near the outer wall of the channel to near the inner wall of the channel. In contrast, the streamlines formed by the focusing of particles of other sizes remained almost unchanged (as shown in Figure 5B). Due to the low flow rate at the beginning, the 25 µm particles were subjected to a small lifting force and a large Dean force, being pushed and being captured by the outfall along with other particles. After the flow rate increased, the enhancement of the Dean force is much smaller than that of the lift force (according to Equations (1) and (2)), so the 25 μm particles are pushed by the lift force to the vicinity of the outer wall of the channel and are collected by the collection port while the other particles are captured by the waste port. Comparing and observing the particle distribution at the inlet and the collection port, as shown in Figure 5C, most 25 μm particles in the sample will be captured and collected by our chip, while 6–15 μm particles will be screened out. The results initially show the performance of our designed chip.

**Figure 6:**
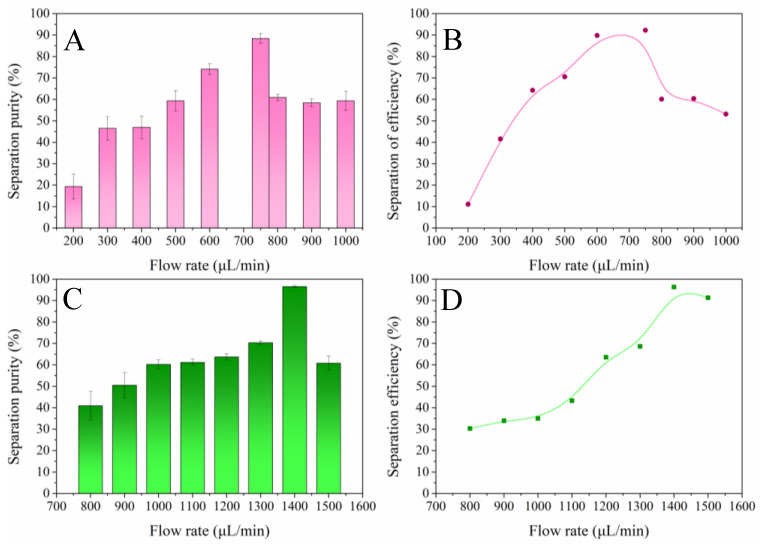
Effect of flow rate on particle sorting efficiency and sorting purity. (**A**). Particle sorting purity of mainstream two-turn rectangular chip; (**B**). particle sorting efficiency of mainstream two-turn rectangular chip; (**C**). particle sorting purity of two-turn trapezoidal spiral chip; (**D**). particle sorting efficiency of two-turn trapezoidal spiral chip.

Using ImageJ software v1.8.0.345 to count particles at the outlet and inlet, the cell separation purity and separation efficiency of the mainstream two-turn rectangular spiral micro-channel chip and our chip were calculated using Equations (9) and (10), and the effect of flow rate on the chip’s separation performance was plotted as shown in Figure 6. By calculating the particle separation effect at the channel mouth, the working performance of the chip designed by the previous authors was obtained (as shown in Figure 6A,B). The general trend indicates enhancement with the increase in flow rate, peaking at 750 μL/min and then gradually declining. The separation performance of the trapezoidal spiral micro-channel chip was calculated and plotted under the same conditions, as shown in Figure 6C,D. The mainstream double-ring chip collected 25 μm particles better at a flow rate of 600–800 μL/min. Its separation purity and efficiency reached the best values at a flow rate of 750 μL/min, achieving 88.6% and 94.1%; our trapezoidal spiral micro-channel chip had better separation performance at a flow rate of 1000–1500 μL/min. The separation purity and efficiency reached their best at 1400 μL/min, with 98.3% and 96.4%, respectively. Our trapezoidal spiral micro-channel chip has better separation performance at flow rates of 1000–1500 μL/min. The chip achieves the best separation purity and efficiency at 1400 μL/min, reaching 98.3% and 96.4%. Based on this result, it can be concluded that our chip has higher cell sorting performance and throughput, taking only about 3.5 min to sort a 5 mL sample. Although there was some deviation between the experiment and simulation due to the randomness and over-idealization of the simulation, as well as the collision of particles during the actual operation, 1400 μL/min was chosen as the optimal initial flow rate.

**Figure 7:**
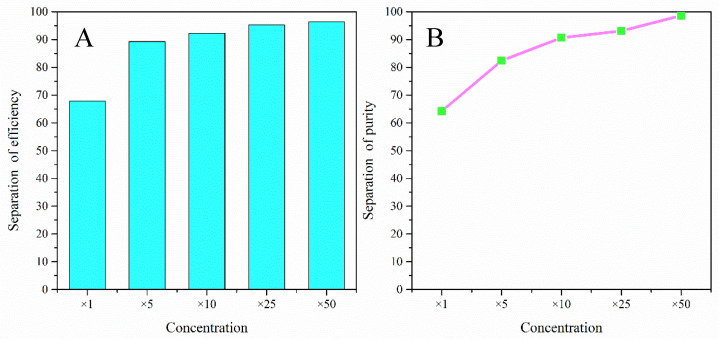
Effect of particle concentration on particle sorting efficiency and sorting purity. (**A**). Particle sorting purity of two-turn trapezoidal spiral chip; (**B**). particle sorting efficiency of two-turn trapezoidal spiral chip.

At the optimal initial flow rate, we continued to explore the effect of particle concentration on sorting. We used a mixture of 6–15 μm and 25 μm polystyrene particles at dilutions of ×5, ×10, ×25, and ×50, respectively. The results of the variation of separation purity and separation efficiency in the dilution ratios are shown in Figure 7A,B. The purity of separation of large particles gradually increases with an increasing dilution ratio because of the fact that the interaction force between particles is large at high concentrations and negligible at low concentrations. After analyzing the results, the separation purity of large particles is optimized when the dilution ratio is greater than or equal to ×50. The separation efficiency of large particles varied with the dilution ratio in line with the separation purity, reaching a maximum when the dilution ratio was equal to ×50. In order to achieve separation efficiency of large particles (target sample) at the collection port, the dilution ratio of the mixed particle solution concentration needs to be set to ×50. The particle content of the solution at this point is about 10^6^ cells/mL.

Comparing the results of simulation and particle simulation experiments (Table 1), we found that the optimal separation flow rate obtained from the actual particle simulation experiments is higher than that obtained from the simulation, but at this time, the difference between the optimal separation efficiency and separation purity is extremely small. With the help of this flow rate shift phenomenon in both chips, we believe that this phenomenon is because the simulation conditions are too idealized to simulate the particle collision in the channel when the chip is actually working. This leads to ignoring the external force generated by the particles colliding with each other when the particle concentration is large. Finally, the results of the simulation experiment can prove that our designed chip has only a slight error in obtaining the best efficiency and purity. These confirm the superior sorting stability of our designed chip.

### 3.3. Cell Sorting Experiments

**Figure 8:**
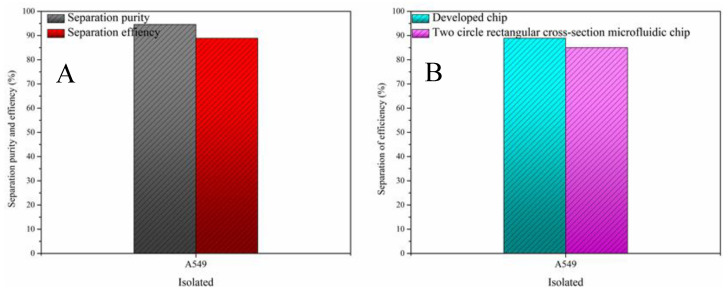
Chip results on A549 cell sorting. (**A**). A549 separation purity and separation efficiency; (**B**). comparison of separation efficiency between our chip and two turns mainstream rectangular micro-channel chip.

After validating the chip using particles, we used A549 cells for further validation. Considering that the concentration of CTCs was very small and inconvenient to observe, we prepared a mixed cell solution of blood cells:CTCs = 1000:1, with a total cell concentration of about 2 × 10^6^ cells/mL, which ensured large background cells and facilitated the observation and recording of subsequent experiments. After passing the sample through the chip, the separation results are shown in Figure 8. The separation efficiency and purity of A549 were 88.9% and 96.4%, respectively. It took only 3.5 min to process a 5 mL sample at a flow rate of 1.4 mL/min, and the high separation performance could be maintained at a lower dilution factor. Meanwhile, we compared the performance of the two-turn rectangular spiral micro-channel chip designed by our predecessor with our own designed chip, demonstrating the superior performance of our designed chip, as shown in Figure 8B. And due to the removal of the sheath fluid, the activity of the isolated A549 cells was more than 94%. This is due to the fact that without the sheath fluid, the particles at the exit are subjected to less crushing impact. Furthermore, a comprehensive comparison of the sorting performance of some of the current passive sorting chips (as shown in Table 2) shows that our chip has a better sorting performance but also has an ultra-fast sample processing speed, which exceeds the effect of most of the passive microfluidic sorting chips. Therefore, the experimental results proved that the trapezoidal spiral micro-channel chip has good performance for the capture of lung cancer CTCs, which can realize high throughput, high sorting efficiency, and convenient operation of sorting.

## 4. Conclusions

In this paper, a microfluidic chip with two-turn trapezoidal spiral micro-channels for CTCs’ enrichment was proposed to achieve the separation of lung cancer tumor cells (A549) from a large number of background cells with a separation efficiency of about 88.9% and 96.4%. By leveraging the spiral and trapezoidal cross-section channels, the integration of these features enabled high-throughput, sheathless, label-free, and high-purity separation of CTCs. The combination of 3D printing and soft lithography not only reduces the process of the chip preparation process and lowers the cost but also facilitates the microscopic observation. The single-entry design reduces operational complexity and avoids the possibility of blockage of the chip entry channel. The designed microfluidic chip with two turns of trapezoidal spiral micro-channels overcomes the previous problem of insufficient flux and easy blockage of the two turns of the chip. By consolidating it to a single inlet, the microfluidic chip with different structures can be further cascaded in the future. This advancement offers a new solution for the simultaneous high-efficiency and high-throughput separation of CTCs of different sizes and a good foundation for the downstream integration of detection methods.

## Figures and Tables

**Table 1:** Comparison of simulation and experimental results of mainstream two-turn rectangular chips and two-turn trapezoidal spiral chips.

Type	Simulation Results	Experimental Results
mainstream two-turn rectangular chips two-turn trapezoidal spiral chips	91.4% (separation efficiency) 98.8% (separation efficiency)	88.6% (separation efficiency) 94.1% (separation purity) 96.4% (separation efficiency) 98.3% (separation purity)

**Table 2:** Performance comparison of different types of current passive sorting microfluidic chips.

Team	Type	Separation Efficiency	Throughput
Ji, H. M. et al. [21]	Microfilter	70~80%	30 μL/min
Warkiani, M. E. et al. [45]	Sprial	~85%	~0.93 μL/min
Warkiani, M. et al. [47]	Sprial	>85%	750 μL/min
Sun et al. [29]	Spiral	96.77%	20 mL/h
Abdulla, A. et al. [46]	Sprial	<80.75%	2 mL/min
Ho, B. D. et al. [23]	DLD	63%	50 μL/min
Fan, L.-L. et al. [24]	elastic	<60%	5 μL/min

## Data Availability

Data are contained within the article.
